# Prognostic value of automated SPECT scoring system for coronary artery disease in stress myocardial perfusion and fatty acid metabolism imaging

**DOI:** 10.1007/s10554-012-0069-6

**Published:** 2012-07-11

**Authors:** Tomoaki Nakata, Akiyoshi Hashimoto, Takayuki Matsuki, Keiichiro Yoshinaga, Kazumasa Tsukamoto, Nagara Tamaki

**Affiliations:** 1 Second Department of Nuclear Medicine, Sapporo Medical University of Medicine, S-1, W-16, Chuo-Ku, Sapporo, Hokkaido 060-8638 Japan; 2 Department of Cardiology, Hokkaido Prefectural Esashi Hospital, Esashi, Japan; 3 Department of Cardiology, Steel Memorial Muroran Hospital, Muroran, Japan; 4 Department of Molecular Imaging, Hokkaido University Graduate School of Medicine, Sapporo, Japan; 5 Nihon Medi-Physics, Co., Ltd., Tokyo, Japan; 6 Department of Nuclear Medicine, Hokkaido University Graduate School of Medicine, Sapporo, Japan

**Keywords:** Automated analysis, Metabolic imaging, Stress perfusion imaging, Risk stratification

## Abstract

Quantitative SPECT analysis contributes to the diagnostic and prognostic assessment of coronary artery disease. A novel automated scoring system (heart score view) can provide identical quantitative information to that determined by expert visual analysis. The aim of the present study is to evaluate the prognostic value of the automated SPECT scoring system when applied to stress thallium and resting beta-methyl-iodophenyl pentadecanoic acid (BMIPP) SPECT images. After a preliminary validation of the automated system by comparison with expert visual analyses, outcome data from 151 consecutive patients with suspected or known coronary artery disease without prior myocardial infarction were analyzed using automated SPECT scores on stress thallium and resting BMIPP images. The software quantified abnormalities as summed stress (SSS), summed rest and summed difference scores for stress thallium and as summed BMIPP scores (SBS). Cardiac events occurred over a period of 48 months in 29 (19.2 %) patients with diabetes mellitus, a lower left ventricular ejection fraction (LVEF) and more abnormal scores for thallium and BMIPP. Multivariate predictors of all cardiac events included diabetes mellitus and thallium SSS. The global Chi-square value was significantly increased when SSS was added to the clinical information (diabetes mellitus and LVEF). Negative predictive values of thallium SSS and SBS were almost identical at 84 % for all cardiac events and 98 % for hard cardiac events. Automatically quantified perfusion and BMIPP scores are related to cardiac events and these values can improve the risk stratification of coronary patients particularly when stress thallium imaging is combined with clinical information.

## Introduction

Together with major clinical risks, stress myocardial perfusion imaging with quantitative assessment has diagnostic and prognostic values for identifying patients at high risk for coronary events [1]. Myocardial perfusion abnormality is visually assessed using summed stress (SSS), summed rest (SRS) and summed difference (SDS) scores in major myocardial perfusion SPECT studies [2–5]. The SPECT scores have been shown to be related to fatal or non-fatal cardiac events [2–5]. The ACNC guidelines recommend the semi-quantitative assessment and further interpretation of myocardial perfusion imaging by experts [6]. However, SPECT analysis is not necessarily a routine procedure in clinical practice at community-based facilities because of a shortage of experts. Standardization of visual assessment requires appropriate training and much experience. Thus, we developed a PC-operated automated scoring system for myocardial SPECT imaging (heart score view software). We previously [7] validated the high reproducibility and accuracy of the automated scoring system for quantitative SPECT analysis when compared with visual expert assessment. Summed SPECT scores were nearly identical between the automated and expert visual assessments on Bland–Altman analysis [7].

Another imaging technique that can be used for the diagnostic and prognostic assessment of coronary patients without stress testing is myocardial fatty acid imaging with ^123^I-beta-methyl-iodophenyl pentadecanoic acid (BMIPP) [8–11]. Myocardial BMIPP imaging has diagnostic and prognostic value for acute and chronic stable coronary artery diseases [8–11]. The automated SPECT software is also applicable to myocardial SPECT imaging with BMIPP. Our earlier study [8] compared the prognostic value of myocardial BMIPP with that of stress thallium imaging in patients with stable coronary artery disease using standard expert visual analysis. However, whether the automated scoring software has prognostic value for myocardial perfusion and BMIPP images remained unknown. Thus, the present study evaluates the prognostic value of the automated SPECT scoring system when applied to stress thallium and resting BMIPP SPECT images of coronary artery disease from this viewpoint. A preliminary study compared automated SPECT scores with semi-quantitative visual scores for prognostic value among 50 patients with coronary disease. This is a statistically acceptable number of patients for analysis using receiver operating characteristics (ROC) curves [12]. Subsequently, the outcomes of all enrolled patients were analyzed using the automated scoring system.

## Methods

### Patient selection

We retrospectively analyzed data from 151 of 196 consecutive patients with suspected or stable coronary artery disease who were originally registered in our database based on the following entry criteria: no history of myocardial infarction, images obtained from resting BMIPP and subsequent stress thallium SPECT imaging, and regular follow up [8]. Data from 45 patients were deleted from outcome analysis based on the following exclusion criteria: loss during follow-up, unknown cause of death, death due to malignancy, coronary revascularization within 3 months of myocardial SPECT imaging and loss of original digital SPECT data files. The Ethics Committee of Shin-Nittetsu Muroran General Hospital approved the study protocol.

### Patient follow-up

The subjects underwent resting BMIPP imaging followed by stress thallium imaging at an interval of 15.8 ± 13.5 (range 2–57) days using a standard protocol and were then followed up to the end points of cardiac death, non-fatal myocardial infarction, late revascularization and hospital admission due to recurrent refractory angina or congestive heart failure. Cardiac death was defined as death attributable to congestive heart failure, myocardial infarction or cardiac arrest or sudden cardiac death. Hard events were defined as cardiac death, non-fatal myocardial infarction and admission due to heart failure.

### Automated quantitative system using heart score view software

We developed the automated software, heart score view, which is a scoring system that is applicable to any type of myocardial SPECT imaging using a standard Windows PC. The automated quantitative method was recently validated by comparison with expert-visual assessment [7]. The software generates a polar-map from myocardial SPECT images then mean % tracer uptake in each segment is automatically scored from normal (0) to absent (4) using a 5-point model based on ASNC guidelines [13]. Thresholds of % tracer uptake for scoring are described elsewhere [7]. Values for SSS, SRS and SDS on stress and rest thallium myocardial perfusion images and summed BMIPP score (SBS) on rest fatty acid image were calculated by summing 17-segment scores on SPECT polar-map images to globally assess SPECT abnormalities. Visual-expert analysis and automated scoring were compared using SPECT images from 50 randomly selected patients with coronary artery disease who were among the 151 patients as a technical validation before starting this outcome study.

### Statistical analysis

Values are expressed as means ± SD. Means of continuous variables were compared using an unpaired *t*-test and categorical data were analyzed using the χ^2^ test. Cardiac event-free survival rates were analyzed using Kaplan–Meier analysis and compared using the log-rank test. Univariable analysis and a subsequent multivariable Cox hazards proportional regression model were applied to identify independent predictive parameters for cardiac events with a hazard ratio and 95 % confidence intervals (CI). Areas under the curve (AUC) among the summed scores were compared using ROC curve analysis. All data were statistically analyzed using MedCalc software, version 9.4.2.0 (Mariakerke, Belgium). A *p* value below 0.05 was considered significant.

## Results

### Preliminary comparison of visual and automated scores in prognosis

Thallium SSS were higher in the non-event group and the AUC in the ROC curve analysis was smaller when assessed by automated SPECT than by expert visual scoring (Tables 1, 2). However, automated SPECT scoring significantly distinguished the event from the non-event group as well as expert visual analysis.

**Table 1:** Preliminary comparison of visual and automated scores for outcome analysis of 50 randomly selected patients

	All cardiac events (n = 13)	No cardiac events (n = 37)	*p* value
Visual-expert analysis			
SSS	6.6 ± 7.5	0.8 ± 1.7*	<0.001
SRS	3.4 ± 6.8	0.4 ± 0.7	0.010
SDS	3.2 ± 4.2	0.5 ± 1.6	0.002
SBS	7.3 ± 7.7	1.2 ± 3.5	0.001
Automated analysis (heart score view)			
SSS	5.7 ± 6.7	1.5 ± 1.8*	0.001
SRS	3.2 ± 5.2	0.9 ± 2.1	0.030
SDS	2.5 ± 2.7	0.6 ± 1.9	0.006
SBS	6.4 ± 7.5	1.9 ± 4.3	0.010

* *p* = 0.002

**Table 2:** Comparison of AUCs using ROC curve analysis obtained by the automated SPECT scoring with visual evaluation

	Visual expert analysis (AUC)	Automated analysis (AUC)	*p* value
SSS	0.838	0.701	0.012
SRS	0.770	0.705	0.455
SDS	0.716	0.689	0.722
SBS	0.789	0.687	0.098

**Fig. 1:**
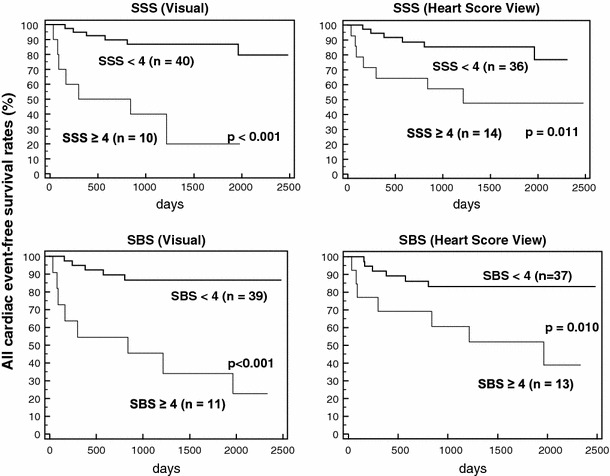
Preliminary comparison of event-free curves between visual expert (*left panels*) and automated SPECT (*right panels*) analyses using cut-off values of SSS and SBS in 50 randomly selected coronary patients

Figure 1 shows that thallium and BMIPP cut-off values derived from the automated scoring system differentiated between high- and low-risk patients as did those determined by expert visual analysis.

### Main outcome study

Twenty-nine cardiac events (7 hard and 22 soft) documented in the 151 patients during a mean follow-up of 48 months were analyzed. One patient had sudden cardiac death, one had cardiac death due to heart failure, two had congestive heart failure, three had non-fatal acute myocardial infarction, eight underwent coronary revascularization at a later stage, and 14 had recurrent angina pectoris requiring hospitalization. Left ventricular ejection fraction (LVEF) was significantly lower and the rate of complication with diabetes mellitus was higher in the group with, than without cardiac events (Table 3). Automated SPECT scores were significantly greater in the group with, than without cardiac events. Likewise, except for SRS, abnormal SPECT scores of ≥4 for thallium SSS, thallium SDS and SBS were more frequent in the group with cardiac events. These results were consistent when only hard cardiac events were considered in the comparison (Table 4).

**Table 3:** Clinical characteristics of 151 patients and all cardiac events

	All cardiac events (n = 29)	No cardiac events (n = 122)	*p* value
Age (years)	64.7 ± 10.4	65.4 ± 10.2	0.745
Gender (male)	19/29 (65.5 %)	64/122(52.5 %)	0.221
Unstable angina pectoris	5/29 (17.2 %)	10/122 (8.2 %)	0.167
LVEF (%)	64.8 ± 10.8	69.7 ± 6.8	0.003
Smoking	10/29 (34.5 %)	39/122 (32.0 %)	0.827
Diabetes mellitus	12/29 (41.4 %)	9/122 (7.4 %)	<0.001
Hypertension	18/29 (62.1 %)	58/122 (47.5 %)	0.215
Dyslipidemia	8/29 (27.6 %)	37/122 (30.3 %)	0.826
ACE inhibitors	4/29 (13.9 %)	16/122 (13.1 %)	1.000
Beta blockers	4/29 (13.9 %)	18/122 (14.8 %)	0.775
Calcium channel blockers	16/29 (55.2 %)	58/122 (47.5 %)	0.537
SSS	3.6 ± 5.5	1.4 ± 2.2	0.001
SRS	1.7 ± 3.8	0.7 ± 1.7	0.031
SDS	1.9 ± 3.5	0.7 ± 1.8	0.012
SBS	3.2 ± 5.8	1.2 ± 3.2	0.030
SSS ≥ 4	10/29 (34.5 %)	18/122 (14.8 %)	0.028
SRS ≥ 4	3/29 (10.3 %)	11/122 (9.0 %)	0.893
SDS ≥ 4	9/29 (31.0 %)	12/122 (9.8 %)	0.008
SBS ≥ 4	8/29 (27.6 %)	13/122 (10.7 %)	0.038

**Table 4:** Clinical characteristics of 151 patients and hard cardiac events

	Hard cardiac events (n = 7)	No hard cardiac events (n = 144)	*p* value
Age (years)	69.7 ± 11.1	65.0 ± 10.1	0.236
Gender (male)	5/7 (71.4 %)	78/144 (54.2 %)	0.459
Unstable angina pectoris	0/7 (0.0 %)	15/144 (10.4 %)	1.000
LVEF (%)	59.3 ± 16.3	69.2 ± 7.1	0.001
Smoking	3/7 (42.9 %)	46/144 (31.9 %)	0.682
Diabetes mellitus	4/7 (57.1 %)	17/144 (11.8 %)	0.008
Hypertension	3/7 (42.9 %)	73/144 (50.7 %)	0.719
Dyslipidemia	0/7 (0.0 %)	45/144 (31.3 %)	0.104
ACE inhibitors	1/7 (14.3 %)	19/144 (13.2 %)	1.000
Beta blockers	2/7 (28.6 %)	21/144 (14.6 %)	0.289
Calcium channel blockers	5/7 (71.4 %)	69/144 (47.9 %)	0.270
SSS	9.4 ± 8.3	1.5 ± 2.2	<0.001
SRS	4.9 ± 6.8	0.7 ± 1.6	<0.001
SDS	4.6 ± 5.2	0.8 ± 1.9	<0.001
SBS	7.3 ± 8.7	1.3 ± 3.3	<0.001
SSS ≥ 4	5/7 (71.4 %)	23/144 (16.0 %)	0.001
SRS ≥ 4	2/7 (28.6 %)	12/144 (8.3 %)	0.256
SDS ≥ 4	4/7 (57.1 %)	17/144 (11.8 %)	0.005
SBS ≥ 4	4/7 (57.1 %)	17/144 (11.8 %)	0.005

### Time dependent analyses using abnormal SPECT scores

**Fig. 2:**
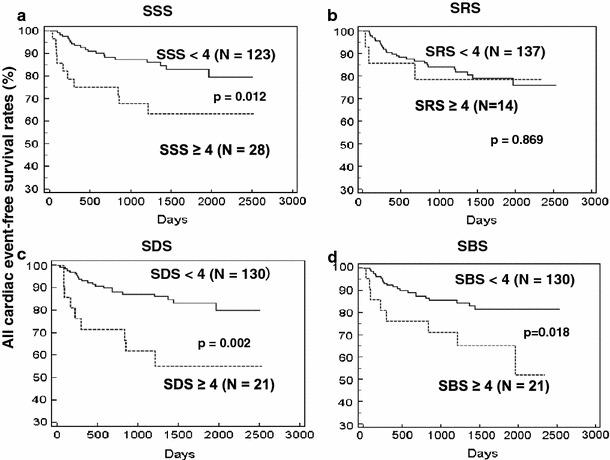
Event-free curves for all cardiac events using SSS, SRS, summed difference SDS and SBS Event-free rates significantly differ between two groups divided by SSS, SDS and SBS but not by SRS. Event-free rates at 2,500 days were 62 and 80 % for patients with SSS ≥ 4 and <4, respectively (**a**), 77 and 75 % with SRS ≥ 4 and <4, respectively (**b**), 55 and 80 % with SDS ≥ 4 and <4, respectively (**c**) and 52 and 82 % with SBS ≥ 4 and <4, respectively (**d**)

**Fig. 3:**
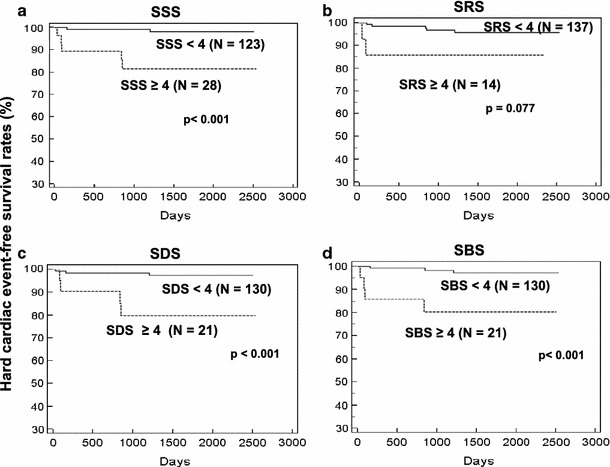
Event-free curves for hard cardiac events using SSS, SRS, SDS and SBS Event-free rates significantly differ between two groups divided by SSS, SDS and SBS but not by SRS. Event-free rates at 2,500 days were 82 and 98 % with SSS ≥ 4 and <4, respectively (**a**), 85 and 94 % with SRS ≥ 4 and <4, respectively (**b**), 80 and 97 % for patients with SDS ≥ 4 and <4, respectively (**c**) and 80 and 97 % with SBS ≥ 4 and <4, respectively (**d**)

Event-free curves were created using the threshold SPECT score of ≥4 that has been established from a 17-segment model as being abnormal [14–19]. Patient subgroups with abnormal scores had significantly lower event free rates for SSS, SDS and SBS when compared with each counterpart for all cardiac events (Fig. 2) and for hard cardiac events (Fig. 3).

### Univariable and multivariable analyses

Univariable Cox analysis revealed LVEF, diabetes mellitus, SSS, SDS and SBS as significant predictors of all cardiac events (Table 5). When four variables identified as clearly significant were included, multivariable Cox analysis uncovered that diabetes mellitus and SSS as significant independent predictors of all cardiac events, whereas LVEF and SBS were not (Table 6). The prognostic value (global Chi-square value) significantly (*p* < 0.01) increased from 21.5 to 31.9 when SSS was added to the clinical information about diabetes mellitus and LVEF.

**Table 5:** Univariate analysis to predict all cardiac events

	Hazard ratio	95 % CI	*p* value
Age (years)	0.994	0.960–1.030	0.740
Gender (male)	0.602	0.281–1.290	0.194
Unstable angina pectoris	2.118	0.812–5.530	0.127
LVEF (%)	0.922	0.882–0.963	<0.001
Smoking	1.166	0.544–2.500	0.695
Diabetes mellitus	6.118	2.926–12.791	<0.001
Hypertension	1.791	0.848–3.782	0.128
Dyslipidemia	0.863	0.384–1.940	0.722
ACE inhibitors	1.081	0.378–3.094	0.885
Beta blockers	1.319	0.505–3.446	0.574
Calcium channel blockers	1.273	0.615–2.638	0.518
SSS	1.184	1.091–1.286	<0.001
SRS	1.103	0.990–1.267	0.069
SDS	1.156	1.034–1.291	0.011
SBS	1.085	1.021–1.153	0.009
SSS ≥ 4	2.578	1.203–5.527	0.015
SRS ≥ 4	1.106	0.336–3.639	0.869
SDS ≥ 4	3.256	1.488–7.129	0.003
SBS ≥ 4	2.572	1.143–5.787	0.023

**Table 6:** Multivariate cox analysis to predict all cardiac events

	Hazard ratio	95 % CI	*p* value
Diabetes mellitus	5.141	2.369–11.156	<0.001
SSS	1.121	1.014–1.239	0.026
SBS ≥ 4	1.139	0.439–2.957	0.789
LVEF	0.958	0.916–1.001	0.059

Global χ^2^, 32.3

### Correlation between stress thallium and BMIPP scores in automated SPECT scoring system

**Fig. 4:**
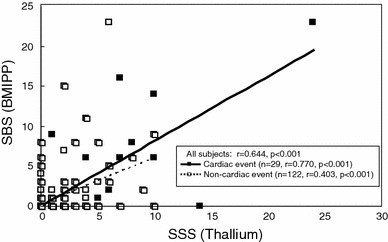
Correlations between SSS and SBS obtained using automated SPECT scoring system

The SBS and SSS significantly correlated in all patients (r = 0.644, *p* < 0.001), as well as in those with (r = 0.770, *p* < 0.001) and without (r = 0.403, *p* < 0.001) cardiac events (Fig. 4).

### Positive and negative predictive values

**Fig. 5:**
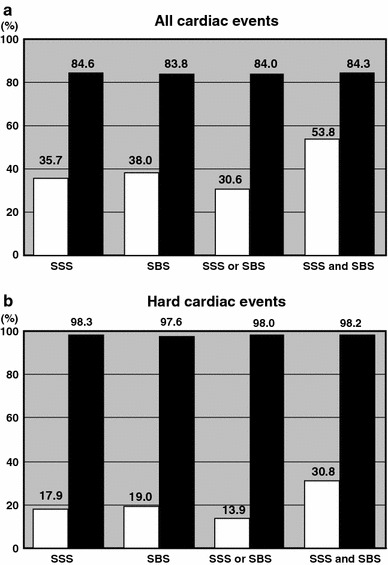
Positive (*white columns*) and negative (*black columns*) predictive values for all (**a**) and hard (**b**) cardiac events using SSS for stress thallium images and/or SBS. Abnormal SSS and SBS are both defined as ≥4; normal scores are defined as ≤3

Figure 5 compares positive and negative predictive values for overall and hard cardiac events when abnormal SSS and SBS were defined as ≥4. Positive predictive values of the abnormal scores were low but increased by combination with SSS and SBS. On the other hand, the negative predictive values of normal SSS and SBS were high and identical without combination at essentially 84 and 98 % for all and hard cardiac events, respectively.

### Case presentation

**Fig. 6:**
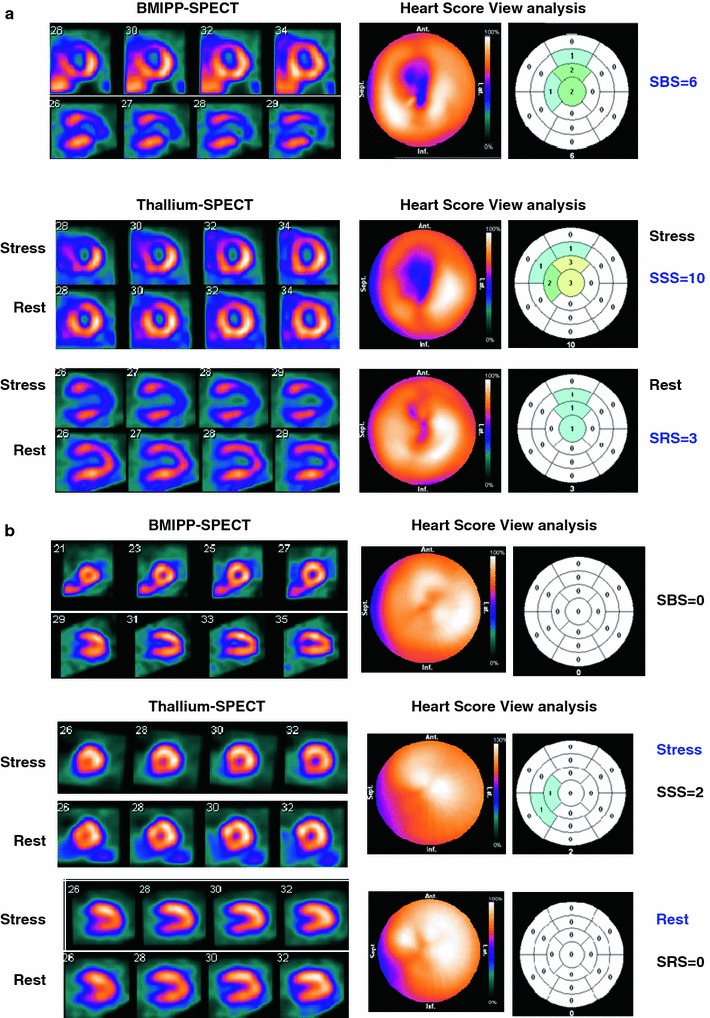
SPECT, polar and heart score view maps of stress and rest thallium myocardial perfusion and rest BMIPP images. **a** Suspected stable coronary artery disease in a 77-year-old hypertensive, diabetic woman. Automated scoring system scored 6, 10 and 3 for BMIPP, stress and rest thallium, respectively, indicating intermediate risk for coronary events. Ninety days later, she underwent emergency percutaneous coronary intervention to treat acute coronary syndrome. **b** Suspected coronary artery disease in a 61-year-old hypertensive, dyslipidemic man with a smoking history. Automated scoring system scored 0, 2 and 0 for BMIPP, stress and rest thallium images, respectively, indicating low risk for cardiac events. He has remained free of cardiac events for 4 years

Figure 6 shows two typical polar-map displays and heart score view maps of rest BMIPP, stress and rest thallium images. A 77-year-old woman with hypertension and diabetes mellitus underwent rest BMIPP followed by exercise-stress thallium imaging due to having suspected stable coronary artery disease. The automated heart score view scores were 6, 10 and 3 for the SBS, SSS and SRS, respectively, indicating an intermediate risk for coronary events. Ninety days later, she underwent emergency percutaneous coronary interventional therapy because of acute coronary syndrome.

A 61-year-old man with a history of smoking, hypertension and dyslipidemia underwent rest BMIPP followed by exercise-stress thallium imaging due to suspected angina pectoris. The automated heart score view system scored 0, 2 and 0 for BMIPP, stress and rest thallium, respectively, indicating a low risk for cardiac events. This patient has remained free of cardiac events for 4 years.

## Discussion

The present findings show that automated SPECT analysis of stress thallium and fatty acid metabolism images can provide reliable, quantitative and additive information for the risk-stratification of patients with coronary artery disease based on high negative predictive values alone and in combination with conventional clinical information such as diabetes mellitus and/or left ventricular function.

### Clinical implications of automated quantitative analysis

Following our previous validation study [7], the current outcome study clarified the prognostic value of automated risk-assessment system using heart score view software in stress thallium and resting metabolic SPECT images in patients with stable coronary artery disease. This is attributable to its high reproducibility, objective and quantitative nature. This PC-operated system can serve as a routine clinical tool for risk stratification, for monitoring therapeutic effects and for creation of large-scale database of an outcome study. Berman et al. [20] reported another type of computer-assisted quantitative analysis using a normal circumferential profile curve in myocardial perfusion SPECT imaging. Although the automated method also used a 5-point scoring system for comparison with standard visual expert analysis, the automated method was not completely identical to the visual scoring system. Expert visual analysis with a 5-point, 17-segment model [1–4] uses three representative short-axis slices at basal, mid-ventricular and apical portions and one vertical-long axis slice. The present automated scoring system uses all short-axis slices derived from SPECT images and count data (% tracer uptake) on polar maps for scoring. In addition, a SPECT abnormality is defined by comparison with a normal SPECT database. Thus, the presented method can provide more global, objective and quantitative information about SPECT images than standard visual analysis. Nevertheless, the present study showed that the prognostic value of the automated scoring system of stress thallium and resting BMIPP SPECT images is as good as that of standard visual analysis.

### Prognostic values of stress thallium and fatty acid uptake abnormalities

As previously demonstrated by visual expert analysis, multivariable analysis in the present study confirmed SSS and diabetes mellitus as powerful independent prognostic determinants. Although SBS and LVEF were significant, they were not necessarily independent variables. Studies including some from Japan have already established the prognostic implications of diabetes mellitus in coronary artery disease [3, 8–10]. Several mechanisms might be involved in the independent correlation between diabetes mellitus and cardiac events found in the present study. Diabetes mellitus is a powerful atherogenic factor that induces or augments coronary sclerosis. The capacity to utilize glucose and fatty acid to generate energy in the diabetic myocardium is reduced, which might lead to cardiac dysfunction. Diabetes mellitus also impairs endothelial vasodilator action and kidney function, which has been recognized as an important determinant of prognosis in cardiac disease [21, 22]. The prognostic power of BMIPP data was not necessarily additive in the present study when stress thallium information (SSS) was included. The discrepant findings of the prognostic value of myocardial BMIPP imaging between the present and our previous [8] study might be attributable to the use of different scoring algorithms. Nevertheless, the present findings clarified that myocardial fatty acid metabolism imaging alone can provide prognostic information without a stress approach. The myocardium produces large amounts of high-energy phosphates by consuming a large amount of oxygen, while it is very vulnerable to ATP depletion [23]. The myocardium can sustain impaired fatty acid uptake for several days to months after acute ischemic events [24–27] or under prolonged ischemia [28–30]. Such metabolic stunning or ischemic memory assessed by BMIPP imaging corresponds to an ischemic risk area that could be responsible for future cardiac events [31, 32]. The present results also revealed that normal SSS and normal SBS had equally high negative predictive values (almost 98 %) for hard cardiac events. Thus, resting myocardial BMIPP values provide additive prognostic information not only for clinically high-risk patients with diabetes mellitus and/or left ventricular dysfunction but also for identifying low-risk patients, particularly as an alternative imaging strategy when stress myocardial perfusion is contraindicated.

### Study limitations

Compared with visual expert analysis in this study, the automated scoring system tended to overestimate thallium SSS and SBS in patients without cardiac events. The automated scoring system in the preliminary study overestimated the SSS in 5 of 37 patients without cardiac events who had heterogeneous hypertrophy or high activity. The discrepancy between the two methods might be due to differences between the scoring systems. The automated system uses all short-axis slices for scoring and quantifies using percent tracer uptake that reflects tracer activity relative to a maximal data point among short-axis slices. Nevertheless, the automated scoring system is unlikely to underestimate the probability of cardiac events in high-risk patients. Automated SPECT analysis is applicable to other types of SPECT imaging. A recent comparison with visual analysis showed that the automated software analysis is technically applicable to ^99m^Tc-tetrofosmin stress myocardial SPECT [33]. Finally, this study retrospectively analyzed myocardial SPECT data from patients without prior myocardial infarction. A future prospective study of a large population is needed to confirm prognostic value of the automated software for the myocardial SPECT imaging of patients with and without prior myocardial infarction.

## Conclusions

Automatically quantified myocardial SPECT abnormality using heart score view software can identify patients at high-risk for coronary artery diseases with high positive and negative predictive values particularly when applied to stress thallium imaging. The automated technique contributes to better prognosis assessment by myocardial SPECT imaging when combined with clinical information such as diabetes mellitus and/or left ventricular function.
